# *Bifidobacterium adolescentis* Isolated from Different Hosts Modifies the Intestinal Microbiota and Displays Differential Metabolic and Immunomodulatory Properties in Mice Fed a High-Fat Diet

**DOI:** 10.3390/nu13031017

**Published:** 2021-03-21

**Authors:** Botao Wang, Qingmin Kong, Shumao Cui, Xiu Li, Zhennan Gu, Jianxin Zhao, Hao Zhang, Wei Chen, Gang Wang

**Affiliations:** 1 State Key Laboratory of Food Science and Technology, Jiangnan University, Wuxi 214122, Jiangsu, China; jnwangbotao@foxmail.com (B.W.); kongqingmin2@163.com (Q.K.); cuishumao@jiangnan.edu.cn (S.C.); lixiu@jiangnan.edu.cn (X.L.); zhennangu@jiangnan.edu.cn (Z.G.); zhaojianxin@jiangnan.edu.cn (J.Z.); zhanghao@jiangnan.edu.cn (H.Z.); chenwei66@jiangnan.edu.cn (W.C.); 2 School of Food Science and Technology, Jiangnan University, Wuxi 214122, Jiangsu, China; 3 International Joint Research Center for Probiotics & Gut Health, Jiangnan University, Wuxi 214122, Jiangsu, China; 4 (Yangzhou) Institute of Food Biotechnology, Jiangnan University, Yangzhou 225004, Jiangsu, China; 5 National Engineering Center of Functional Food, Jiangnan University, Wuxi 214122, Jiangsu, China; 6 Translational Medicine Research Center and Jiangsu Translational Medicine Research Institute Wuxi Branch, Wuxi 214122, Jiangsu, China

**Keywords:** high-fat diet, *Bifidobacterium adolescentis*, energy metabolism, immunity, intestinal microbiota, short-chain fatty acids

## Abstract

The incidence of obesity, which is closely associated with the gut microbiota and chronic inflammation, has rapidly increased in the past 40 years. Therefore, the probiotic-based modification of the intestinal microbiota composition has been developed as a strategy for the treatment of obesity. In this study, we selected four *Bifidobacterium adolescentis* strains isolated from the feces of newborn and elderly humans to investigate whether supplementation with *B. adolescentis* of various origins could alleviate obesity in mice. Male C57BL/6J mice fed a high-fat diet (HFD, 60% energy as fat) received one of the following 14-week interventions: (i) *B. adolescentis* N4_N3, (ii) *B. adolescentis* Z25, (iii) *B. adolescentis* 17_3, (iv) *B. adolescentis* 2016_7_2, and (v) phosphate-buffered saline. The metabolic parameters, thermogenesis, and immunity of all treated mice were measured. Cecal and colonic microbial profiles were determined by 16S rRNA gene sequencing. Intestinal concentrations of short-chain fatty acids (SCFAs) were measured by gas chromatography-mass spectrometry (GC-MS). The *B. adolescentis* strains isolated from the feces of elderly humans (*B. adolescentis* Z25, 17_3, and 2016_7_2) decreased the body weight or weight gain of mice, whilst the strain isolated from the newborn (*B. adolescentis* N4_N3) increased the body weight of mice. The *B. adolescentis* strains isolated from the elderly also increased serum leptin concentrations and induced the expression of thermogenesis- and lipid metabolism-related genes in brown adipose tissue. All the *B. adolescentis* strains alleviated inflammations in the spleen and brain and modified the cecal and colonic microbiota. Particularly, all strains reversed the HFD-induced depletion of *Bifidobacterium* and reduced the development of beta-lactam resistance. In addition, the *B. adolescentis* strains isolated from the elderly increased the relative abundances of potentially beneficial genera, such as *Bacteroides*, *Parabacteroides,* and *Faecalibaculum*. We speculate that such increased abundance of commensal bacteria may have mediated the alleviation of obesity, as *B. adolescentis* supplementation decreased the intestinal production of SCFAs, thereby reducing energy delivery to the host mice. Our results revealed that certain strains of *B. adolescentis* can alleviate obesity and modify the gut microbiota of mice. The tested strains of *B. adolescentis* showed different effects on lipid metabolism and immunity regulation, with these effects related to whether they had been isolated from the feces of newborn or elderly humans. This indicates that *B. adolescentis* from different sources may have disparate effects on host health possibly due to the transmission of origin-specific functions to the host.

## 1. Introduction

The development of economic globalization has led to great improvements in the quality of life of many people worldwide. This has caused changes in dietary patterns leading to excessive nutrition intake, and a consequent increase in the prevalence of obesity. Data from the World Health Organization (WHO) in 2016 revealed that the number of overweight adults had exceeded 1.9 billion and that more than 34% of these adults were obese. In addition, in 2019 more than 340 million children aged 5–19 years were overweight or obese, and 38 million children aged under 5 years were overweight or obese [1]. Obesity is therefore a global health problem as it can lead to the development of various diseases [2,3].

The western diet is characterized by a high consumption of meat, fat, and deep-processed, fiber-deficient carbohydrates [4]. In recent decades, consumption of the western diet has become increasingly commonplace throughout the world. This alteration of dietary habits has increased rates of obesity and affected the gut microbiota, which has increased the prevalence of metabolic diseases [5,6]. Studies with germ-free mice have confirmed that the gut microbiota is closely associated with obesity [7], and its alteration is an effective remedial treatment for obesity [8,9]. Furthermore, the consumption of an HFD causes gut microbiota disorders, accompanied by a reduction in the intestinal abundance of the genus *Bifidobacterium* [10], which plays important roles in intestinal health, energy metabolism, and immunity [11,12]. To reverse this HFD-induced suppression of *bifidobacteria*, supplementation with *bifidobacteria* and/or prebiotics has been explored as a treatment for animal models of obesity and it has been revealed that some strains of *bifidobacteria* can alleviate obesity [12,13]. 

We have previously shown that *Bifidobacterium adolescentis* alleviates metabolic disorders and non-alcoholic fatty liver disease [14,15]. Therefore, in this study, we selected four *B. adolescentis* strains isolated from healthy human feces and evaluated their effects on HFD-induced obesity in mice. We also compared the effects of these strains on lipid metabolism, inflammation, intestinal microbiota composition, and bacteria-generated SCFAs to determine how *B. adolescentis* supplementation alleviates obesity in mice.

## 2. Materials and Methods

### 2.1. B. adolescentis Strains and Culture

*B. adolescentis* 17_3, Z25, N4_N3, and 2016_7_2 were acquired from the Research Center of Food Biotechnology of Jiangnan University. These *B. adolescentis* strains had previously been isolated from the feces of healthy humans from different provinces of China for public-health research purposes (Table 1). The use of the collected samples was approved by the participants or their legal guardians. *B. adolescentis* strains were cultured in De Man, Rogosa, and Sharpe medium (MRS, containing 0.05% cysteine) at 37 °C in an anaerobic workstation.

### 2.2. Animals and Experimental Design

This project was approved by the Ethics Committee of Experimental Animals at Jiangnan University (qualified number: JN.No20181230c0880715 (284)). Male C57BL/6J (4–5-week-old) mice were purchased from Gempharmatech Co., Ltd. (Nanjing, China) and housed in an SPF-grade laboratory animal facility under a standard 12 h:12 h light:dark cycle with free access to water and food. After 1-week adaption with a normal-chow diet, the mice were divided into five groups (n = 6 per group) and each group was fed a refined HFD diet (60% fat, TP23300, TROPHIC, Nantong, China). The diet composition is shown in Table S1. Four groups were supplemented once daily for 14 weeks with different *B. adolescentis* strains via oral gavage of 0.2 mL PBS containing approximately 2 × 10^8^ colony-forming units, while the control group was treated with 0.2 mL PBS via oral gavage once daily for 14 weeks (HFD group). The HFD was freshly weighed and dispensed every second day. The body weight of the mice was recorded twice weekly. The cages were replaced twice a week to maintain a clean environment. At the end of the experiment, the mice were killed by euthanasia after being fasted for 12 h [16]. Fresh blood was collected, left to stand for 2 h, and then centrifuged (at 1000× *g* and 4 °C for 15 min) to isolate serum. The liver, spleen, abdominal white adipose tissue (aWAT), and interscapular brown adipose tissue (BAT) were isolated and weighed. These tissues and subcutaneous white adipose tissue (sWAT), brain tissue, and whole intestinal tracts were put into liquid nitrogen and then stored at –80 °C until analysis.

### 2.3. Biochemical Measurements

Serum biochemical indices total cholesterol (TC), low-density lipoprotein cholesterol (LDL-C), high-density lipoprotein cholesterol (HDL-C), triacylglycerol (TG), glucose, alanine aminotransferase (ALT), and aspartate aminotransferase (AST)] were determined using a Mindray biochemical analyzer (Mindray, Shenzhen, China).

### 2.4. Serum Leptin and Glucagon-Like Peptide-1(GLP-1) Concentrations

Serum leptin (LEP)and glucagon-like peptide 1 (GLP-1) concentrations were quantified using a mouse LEP enzyme-linked immunosorbent assay (ELISA) kit(E-EL-M3008) and a mouse GLP-1 ELISA kit (E-EL-M0090c), according to the manufacturer’s instructions (Elabscience Biotechnology Co., Ltd., Wuhan, China).

### 2.5. Metabolic Rate Analyses

Metabolic rates were analyzed using a Comprehensive Lab Animal Monitoring System (Columbus Instruments), according to Sungsoon et al. [17]. 

### 2.6. Gene Expression Analysis

Total RNA of tissues was extracted with TRIzol reagent (15596026, Invitrogen, Waltham, MA, USA). cDNA was synthesized using a reverse transcription kit purchased from CoWin Biosciences (Beijing, China). A quantitative real-time polymerase chain reaction (PCR) was performed in a BioRad thermocycler using the qPCR Master Mix (Vazyme Biotech Co., Ltd, Nanjing, China) and the primer sequences listed in Table S2. PCR programs and data analyses were based on the previous study [16].

### 2.7. Quantification of Cytokines in Brain Tissue and aWAT

Tissue cytokines were quantified using a mouse ELISA DuoSet kit (R&D Systems, Minneapolis, MN, USA) according to the manufacturer’s instructions, including IL-6 (DY406), IL-10 (DY417), and IL-17(A) (DY421).

### 2.8. Gut Microbiota Sequencing

The total DNA of the cecal and colonic microbiota was extracted and used for PCR amplification of the V3–V4 regions of their respective 16S rRNA genes. Amplicon sequencing, sequence analysis, and function prediction were performed according to the previous study [16]. The sequencing reads were processed by the QIIME2 plugin DADA2. Taxonomic classification was processed by the q2-feature-classifier plugin using the Silva reference database (silva-132-99-nb-341F-806R-classifier.qza). These analyses were undertaken using QIIME 2 2019.7. Alpha and beta diversity of the microbiome data were analyzed by using the pipeline action core-metrics-phylogenetic on QIIME 2. Differences in the alpha diversity between groups were analyzed by using the pipeline action alpha-group-significance on QIIME2 with Kruskal–Wallis pairwise test for multiple comparisons. Beta diversity was measured by Bray–Curtis distance, and differences in the beta diversity between groups were analyzed using the pipeline action beta-group-significance on QIIME2 with pairwise PERMANOVA. Differentiate features were identified by ANCOM [18] and Linear discriminant analysis (LDA) effect size (LEfSe). LEfSe was visualized by taxonomic cladogram tree and abundance histograms (Kruskal–Wallis test and Wilcoxon rank-sum test, α < 0.05 and logLDA > 2.0 were used as thresholds). 

### 2.9. Quantification of SCFAs in Intestinal Contents

To quantify SCFAs in cecal and colonic contents, fresh intestinal contents were freeze-dried before weighing, and then SCFAs were quantified as reported in the previous study [16].

### 2.10. Statistical Analysis

Data are exhibited as means ± standard deviations (SD), and one-way analysis of variance (ANOVA) was performed to compare the effects of *B. adolescentis* strains in mice fed the HFD, followed by Dunnett’s multiple comparisons test against the HFD group. Data analysis was performed on a GraphPad Prism 6 (GraphPad Software Inc., San Diego, CA, USA), Statistical Package for the Social Sciences 19.0 (SPSS Inc., Chicago, IL, USA), and STAMP. Principal component analysis (PCA) was performed using a SIMICA 14 (Umetrics, Umeå, Sweden). The linear discriminant analysis effect size (LEfSe) was determined on a Galaxy/Hutlab platform.

## 3. Results

### 3.1. B. adolescentis Has Different Metabolic Effects on Mice Fed an HFD

**Figure 1:**
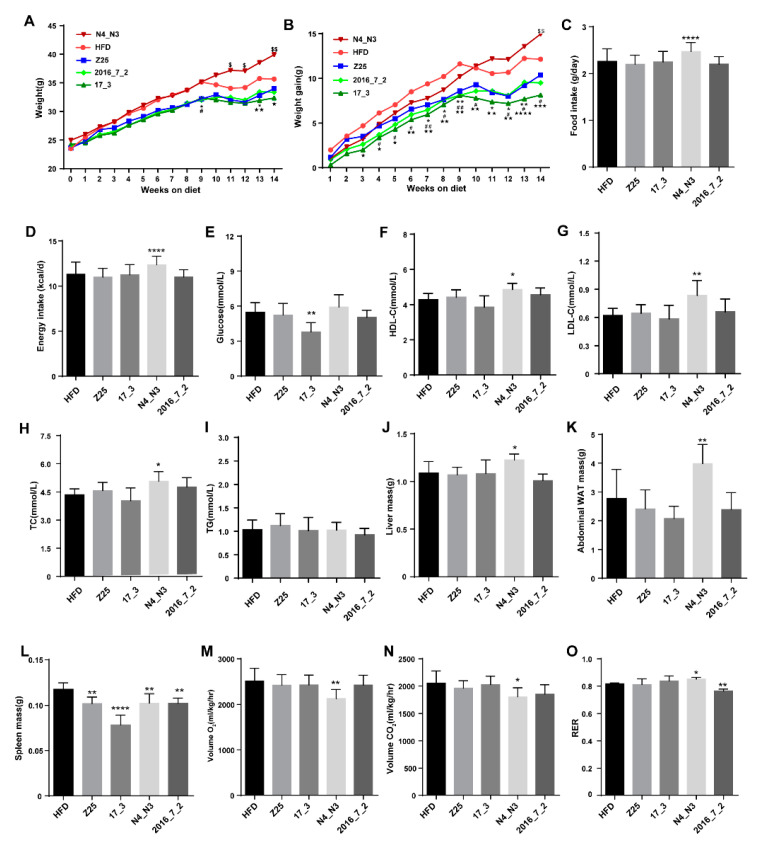
Effects of *B. adolescentis* supplementations on mouse physiology. (**A**) Mean weekly body weight of mice. (**B**) Mean weekly body weight gain of mice. (**C**) Daily food intake of mice. (**D**) Daily energy intake of mice. (**E**–**I**) Concentrations of blood biochemical indices, namely glucose, high-density lipoprotein cholesterol (HDL-C), low-density lipoprotein cholesterol (LDL-C), total cholesterol (TC), and triacylglycerol (TG). (**J**–**L**) Tissue mass of mice, (**J**) liver mass, (**K**) abdominal white adipose tissue, and (**L**) spleen mass. (**M**–**O**) Metabolic rates of mice, (**M**) consumption of O_2_, (**N**) production of CO_2_, and (**O**) respiratory exchange ratio (RER). Data are shown as means ± standard deviations (SD). Markers indicate significant differences (one-way ANOVA, * *p* < 0.05, ** *p* < 0.01, **** *p* < 0.0001; A and B: $ *p* < 0.05, $$ *p* < 0.01, N4_N3 vs. HFD; * *p* < 0.05, Z25 vs. HFD; **#** *p* < 0.05, **##** *p* < 0.01, 2016_7_2 vs. HFD; ★ *p* < 0.05, ★★ *p* < 0.01, ★★★ *p* < 0.001, ★★★★ *p* < 0.0001, 17_3 vs. HFD, *n* = 6 mice per group).

An HFD clearly increased the body weight of the mice. However, *B. adolescentis* intervention altered the body weight and weight gain of the mice. In particular, compared to the body weight of the mice in the HFD group, *B. adolescentis* N4_N3 supplementation significantly increased the body weight of mice, while *B. adolescentis* 17_3 supplementation decreased the body weight of mice, with the body weight of the *B. adolescentis* 17_3 group being lower than that of the HFD group (Figure 1A). The body weights in the *B. adolescentis* 2016_7_2 and *B. adolescentis* Z25 supplementation groups tended to be lower than that of the HFD group, but the difference was not significant. The body weight gain of mice treated with *B. adolescentis* strains isolated from the elderly was significantly reduced over the first 9 weeks of treatment, compared to the HFD group. In addition, at the 14th week and compared to the HFD group, it was found that *B. adolescentis* N4_N3 supplementation had significantly increased the weight gain of mice, but *B. adolescentis* 2016_7_2 and 17_3 supplementation had significantly decreased the weight gain of mice (Figure 1B). No difference in food and energy intake was observed between the *B. adolescentis* 17_3 and 2016_7_2, Z25 supplementation groups and the HFD group, but this intake was significantly increased in the *B. adolescentis* N4_N3 supplementation group (Figure 1C,D). In addition, the *B. adolescentis* 17_3 supplementation group had a lower fasting blood-glucose concentration than the HFD group (Figure 1E), while the *B. adolescentis* N4_N3 supplementation group had higher concentrations of serum HDL-C, LDL-C, and TC than the HFD group (Figure 1F–H). Tissue weights also varied between the HFD and *B. adolescentis* supplementation groups. The *B. adolescentis* N4_N3 supplementation group had a higher liver mass (*p* = 0.0255, Figure 1J) and a higher aWAT mass (*p* = 0.006, Figure 1K) than the HFD group. All *B. adolescentis* supplementation groups had significantly decreased spleen mass compared with the HFD group (HFD vs. Z25: *p* = 0.004; HFD vs. 17-3: *p* < 0.0001; HFD vs. N4_N3, *p* = 0.0048; HFD vs. 2016-7-2: *p* = 0.0047; Figure 1L). 

Based on the above results, we speculated that *B. adolescentis* supplementation might alter the metabolic rates of the HFD-fed mice. We therefore performed a metabolic rate analysis, which revealed that O_2_ consumption and CO_2_ production in the *B. adolescentis* N4_N3 supplementation group was lower than that in the HFD group (O_2_ consumption: *p* = 0.0083, Figure 1M; CO_2_ production: *p* = 0.0206, Figure 1N). Furthermore, the respiratory exchange ratio (RER) of the *B. adolescentis* N4_N3 supplementation group was notably higher than that of the HFD group, indicating that *B. adolescentis* N4_N3 supplementation reduced the metabolic rates of the HFD-fed mice, such that they consumed less fat for energy metabolism compared with the HFD group. Although there was no difference observed in O_2_ consumption and CO_2_ generation between the HFD group and the groups treated with the *B. adolescentis* strains isolated from the elderly (Figure 1M,N), *B. adolescentis* 2016_7_2 significantly decreased the respiratory exchange ratio (RER) of the HFD-fed mice (*p* = 0.0018, Figure 1O), which implies that the *B. adolescentis* 2016_7_2 supplementation group might have consumed more fat for energy metabolism than the HFD group (O_2_ consumption, CO_2_ production, and RER of mice in light and dark were shown in Figure S1).

### 3.2. B. adolescentis Supplementation Regulated Lipid Metabolism in the BAT, Liver, and sWAT

The metabolic rate analysis revealed that *B. adolescentis* supplementation altered the energy metabolism of the HFD-fed mice, which was associated with lipid metabolism. Therefore, lipid metabolism in the BAT, liver tissue, and sWAT was evaluated.

**Figure 2:**
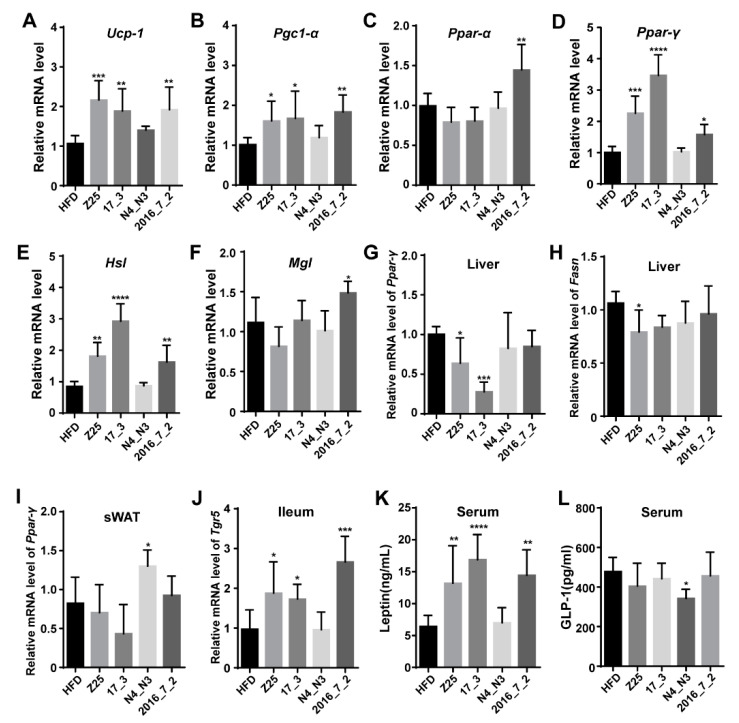
Effects of *B. adolescentis* supplementations on energy metabolism. (**A**–**D**) Relative mRNA expression of thermogenesis genes uncoupling protein 1 (*Ucp-1*), peroxisome proliferator-activated receptor-γ coactivator-1α (*Pgc1-α*), peroxisome proliferator-activated receptor-alpha (*Ppar-α*), and peroxisome proliferator-activated receptor-gamma (*Ppar-γ*) in the brown adipose tissue (BAT). (**E**,**F**) Relative mRNA expression of lipolytic enzyme genes hormone-sensitive lipase (*Hsl*) and acylglycerol lipase (*Mgl*) in the BAT. (**G**) Relative mRNA expression of *Ppar-γ* in the liver. (**H**) Relative mRNA expression of fatty acid synthase (*Fasn*) in the liver. (**I**) Relative mRNA expression of *Ppar-γ* in the subcutaneous white adipose tissue (sWAT). (**J**) Relative mRNA expression of the bile acid receptor *Tgr5* in the ileum. (**K**,**L**) Concentrations of serum leptin and glucagon-like peptide-1 (GLP-1). Data are shown as means ± standard deviations (SD). Asterisks indicate significant differences (one-way ANOVA, * *p* < 0.05, ** *p* < 0.01, *** *p* < 0.001, **** *p* < 0.0001). *n* = 5 or *n* = 6 (for **K**,**L**) mice per group.

Given the RER results, the mRNA expression levels of genes associated with thermogenesis and lipid metabolism were analyzed in the BAT. The mRNA expression levels of the thermogenesis-related genes uncoupling protein 1 (*Ucp-1*) and peroxisome proliferator-activated receptor (PPAR) gamma coactivator 1-alpha–encoding (*Pgc1-α*) were found to be significantly upregulated in the BAT derived from the *B. adolescentis* Z25, *B. adolescentis* 17_3, and *B. adolescentis* 2016_7_2 supplementation groups compared with those in the HFD group, while there was no change in the expression levels of these genes in the *B. adolescentis* N4_N3 supplementation group (*Ucp-1*: HFD vs. Z25, *p* = 0.0006; HFD vs. 17_3, *p* = 0.007; HFD vs. 2016_7_2: *p* = 0.0052; *Pgc1-α*: HFD vs. Z25: *p* = 0.049; HFD vs. 17_3: *p* = 0.0308; HFD vs. 2016_7_2: *p* = 0.0066, Figure 2A,B). Moreover, PPAR alpha and gamma are nuclear receptor proteins encoded by the genes *Ppar-α* and *Ppar-γ*, which play key roles in lipid metabolism. Compared with the HFD group, the *B. adolescentis* 2016_7_2 supplementation group displayed a significantly increased expression of *Ppar-α* mRNA in the BAT (*p* = 0.0035, Figure 2C). In addition, *Ppar-γ* mRNA expression in the BAT of the *B. adolescentis* Z25, 17_3 and 2016_7_2 supplementation groups was higher by 2.4-, 3.4-, and 1.6-fold, respectively, than that in the BAT of the HFD group (HFD vs. Z25: *p* = 0.0001; HFD vs. 17_3: *p* < 0.0001; HFD vs. 2016_7_2: *p* = 0.0439, Figure 2D). The mRNA expression of two lipolytic enzymes—hormone-sensitive lipase (HSL) and acylglycerol lipase (MGL)—in the BAT was also altered by *B. adolescentis* supplementation. The *Hsl* mRNA level in the BAT of the *B. adolescentis* Z25, *B. adolescentis* 17_3, and *B. adolescentis* 2016_7_2 supplementation groups was higher by 2.1-, 3.4-, and 1.9-fold, respectively, than that in the BAT of the HFD group (HFD vs. Z25: *p* = 0.0012; HFD vs. 17_3: *p* < 0.0001; HFD vs. 2016_7_2: *p* = 0.0065, Figure 2E). Similarly, the *Mgl* mRNA level in the *B. adolescentis* 2016_7_2 supplementation group was significantly higher than that in the HFD group (*p* = 0.027, Figure 2F).

In the liver, the *Ppar-γ* mRNA expression levels in the *B. adolescentis* Z25 and 17_3 supplementation groups were notably lower than that in the HFD group (HFD vs. Z25: *p* = 0.0464; HFD vs. 17_3: *p* = 0.0004, Figure 2G). However, there was no difference in the *Ppar-α*, *Pgc1-α*, *Hsl*, and *Mgl* mRNA expression levels between the HFD and *B. adolescentis* supplementation groups (Figure S2A). The expression of the fatty acid synthase (FAS)-coding gene (*Fasn*) mRNA was also significantly downregulated in the *B. adolescentis* Z25 supplementation group compared with that in the HFD group (*p* = 0.0336, Figure 2H). 

In the sWAT, the expression level of *Ppar-γ* mRNA was higher in the *B. adolescentis* N4_N3 supplementation group than in the HFD group (*p* = 0.0257, Figure 2I). However, there was no statistically significant difference in the expression of *Ppar-α*, *Pgc1-α*, *Hsl*, *Mgl*, and Prdm 16 mRNA between the HFD and the *B. adolescentis* supplementation groups (Figure S2B). 

The above results showed that *B. adolescentis* supplementation notably altered lipid metabolism-related genes, especially in thermogenesis and lipidolysis in the BAT and lipid synthesis in the liver and sWAT, whereas the nature of this alteration differed according to the *B. adolescentis* strain that was used. Therefore, we speculated that *B. adolescentis* supplementation might trigger the expression of the receptors involved in the induction of thermogenesis and lipid metabolism in the intestine. In addition, there were significant differences in the serum concentrations of HDL-C, LDL-C, and TC between these five groups, which implied that *B. adolescentis* supplementation affected bile acid (BA) function. Thus, the mRNA expression levels of genes coding for the BA receptors farnesoid X receptor (FXR) and G protein-coupled bile acid receptor 1 (Gpbar 1, also known as GPR131 or TGR5) were measured in the ileum tissues of mice. There was no significant difference in the expression level of *Fxr* mRNA between any of the groups (Figure S2C), but the *B. adolescentis* Z25, 17_3 and *B. adolescentis* 2016_7_2 supplementation groups exhibited notably upregulated levels of *Tgr5* mRNA in the ileum compared with the HFD group, consistent with the upregulated expression of the mRNA of the thermogenesis genes *Ucp-1* and *Pgc1-α* in the BAT (HFD vs. Z25: *p* = 0.0198; HFD vs. 17_3: *p* = 0.0479; HFD vs. 2016_7_2: *p* = 0.0001, Figure 2J).

In addition, there were significant differences in the serum concentrations of the hormones leptin and GLP-1 between the HFD supplementation group and the *B. adolescentis* supplementation groups (Figure 2K,L). The *B. adolescentis* Z25, *B. adolescentis* 17_3, and *B. adolescentis* 2016_7_2 supplementation groups had significantly higher serum leptin concentrations than the HFD group (HFD vs. Z25: *p* = 0.0054; HFD vs. 17_3: *p* < 0.0001; HFD vs. 2016_7_2: *p* = 0.0014, Figure 2K), and the *B. adolescentis* N4_N3 supplementation group had lower serum GLP-1 concentrations than the HFD group (*p* = 0.0154, Figure 2L).

### 3.3. B. adolescentis Supplementation Alleviates Inflammation Induced by the HFD

**Figure 3:**
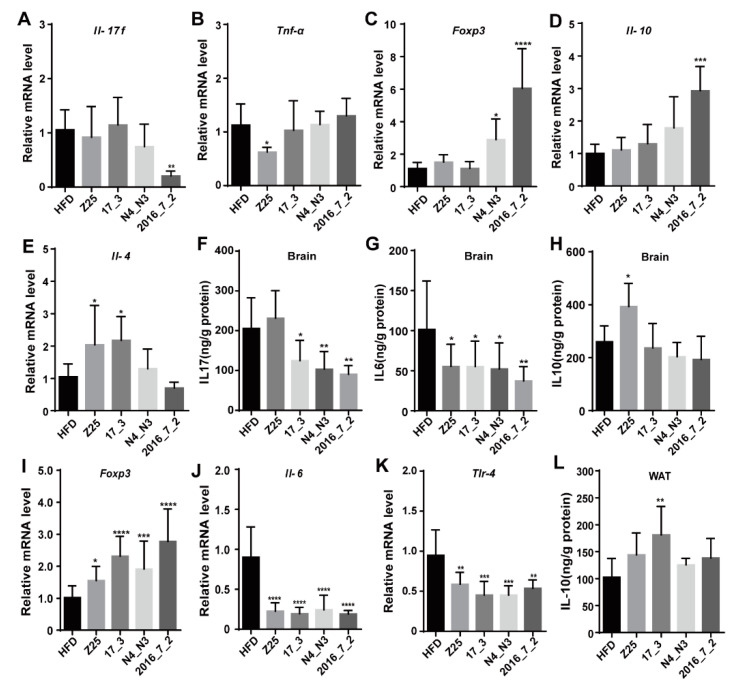
Effects of *B. adolescentis* supplementations on immunity. (**A**–**E**) Relative mRNA expression of immune-related cytokines in the spleen, (**A**) interleukin 17F (*Il-17f*), (**B**) tumor necrosis factor α (*Tnf-α*), (**C**) forkhead box P3 (*Foxp3*), (**D**) *Il-10,* and (**E**) *Il-4*. (**F**–**H**) Concentrations of cytokines in the brain, (**F**) IL-17, (**G**) IL-6, and (**H**) IL-10. (**I**–**K**) Relative mRNA expression of immune-related cytokines in the hypothalamus, (**I**) *Foxp3*, (**J**) *Il-6*, and (**K**) toll-like receptor 4 (*Tlr4*). (**L**) The concentration of cytokine IL-10 in the abdominal white adipose tissue (aWAT). Data are shown as means ± standard deviations (SD). Asterisks indicate significant differences (one-way ANOVA, * *p* < 0.05, ** *p* < 0.01, *** *p* < 0.001, **** p < 0.0001). *n* = 6 or *n* = 5 (for (**A**–**E**,**I**–**K**)) mice per group.

Due to the differences in spleen weight between the HFD supplementation group and the *B. adolescentis* supplementation groups, we speculated that there were differences in inflammation between these groups. Therefore, we determined the expression levels of mRNA of immune-related cytokines in the spleen. Compared with the HFD group, the *B. adolescentis* 2016_7_2 supplementation group exhibited notably lower levels of interleukin 17F (*Il-17f*) mRNA (*p* = 0.0047, Figure 3A), while the *B. adolescentis* Z25 supplementation group exhibited lower levels of tumor necrosis factor α (*Tnf-α*) mRNA (*p* = 0.0361, Figure 3B). However, there was no difference in the expression level of toll-like receptor 4 (*Tlr4*) mRNA between the HFD group and the *B. adolescentis* supplementation groups (Figure S3A). Nevertheless, *B. adolescentis* supplementation did significantly upregulate the expression of anti-inflammatory cytokines. The *B. adolescentis* N4_N3 and *B. adolescentis* 2016_7_2 supplementation groups exhibited higher levels of forkhead box P3 (*Foxp3*) mRNA expression than the HFD group (HFD vs. N4_N3: *p* = 0.0387; HFD vs. 2016-7-2: *p* < 0.0001, Figure 3C). Moreover, the *B. adolescentis* 2016_7_2 supplementation group exhibited significantly higher levels of *Il-10* mRNA expression (*p* = 0.0001, Figure 3D) than the HFD group, and the levels of *Il-4* mRNA expression in the *B. adolescentis* Z25 and *B. adolescentis* 17_3 supplementation groups were higher than that in the HFD group (HFD vs. Z25: *p* = 0.0430; HFD vs. 17_3: *p* = 0.0227, Figure 3E).

Studies have reported that obesity caused by diets can cause inflammation, including in peripheral tissues and the brain [19,20]. The hypothalamus is the core controller of energy metabolism, and obesity is associated with hypothalamic injury [20]. Given these immunity-related effects in the spleen, we further explored whether the inflammation of the brain, especially the hypothalamus, was improved by *B. adolescentis*. Surprisingly, compared with the HFD group, the *B. adolescentis* 17_3, *B. adolescentis* N4_N3, and *B. adolescentis* 2016_7_2 supplementation groups exhibited significantly lower brain concentrations of IL-17 (HFD vs. 17-3: *p* = 0.019; HFD vs. N4-N3: *p* = 0.0041; HFD vs. 2016-7-2: *p* = 0.0015, Figure 3F), and all *B. adolescentis* supplementation groups showed lower brain concentrations of IL-6 (HFD vs. Z25: *p* = 0.0414; HFD vs. 17-3: *p* = 0.0407; HFD vs. N4-N3: *p* = 0.0256; HFD vs. 2016-7-2: *p* = 0.0061, Figure 3G). In addition, the concentration of IL-10 in the brains of the *B. adolescentis* Z25 supplementation group was notably higher than that in the brains of the HFD group (*p* = 0.0326, Figure 3H). 

Furthermore, the expression of immune-related cytokines in the hypothalamus was measured. All *B. adolescentis* supplementation groups exhibited significantly upregulated levels of *Foxp3* mRNA compared with the HFD group (HFD vs. Z25: *p* = 0.0432; HFD vs. 17-3: *p* < 0.0001; HFD vs. N4-N3: *p* = 0.0008; HFD vs. 2016-7-2: *p* < 0.0001, Figure 3I). However, the level of *Il-6* mRNA in the *B. adolescentis* supplementation groups was much lower than that in the HFD group (HFD vs. Z25: *p* < 0.0001; HFD vs. 17-3: *p* < 0.0001; HFD vs. N4-N3: *p* < 0.0001; HFD vs. 2016-7-2: *p* < 0.0001, Figure 3J), as was the level of Tlr 4 mRNA concentration (HFD vs. Z25: *p* = 0.0063; HFD vs. 17-3: *p* = 0.0004; HFD vs. N4-N3: *p* = 0.0004; HFD vs. 2016-7-2: *p* = 0.0024, Figure 3K). No statistically significant differences were found in the levels of *Il-4*, *Tnf-α,* and *Il-1β* mRNA between the HFD and the *B. adolescentis* supplementation groups (Figure S3B–D).

The concentrations of cytokines in the aWAT was also determined. The concentration of IL-10 in the aWAT of the *B. adolescentis* 17_3 supplementation group was significantly higher than that in the aWAT of the HFD group (*p* = 0.0015, Figure 3L). However, no statistically significant differences were found in the concentrations of TNF-α and IL-1β in the aWAT between the HFD group and the *B. adolescentis* supplementation groups (Figure S3E,F).

### 3.4. B. adolescentis Supplementation Alters the Gut Microbiota Composition in the Cecum and Colon

#### 3.4.1. Gut Microbiota Composition in the Cecum

**Figure 4:**
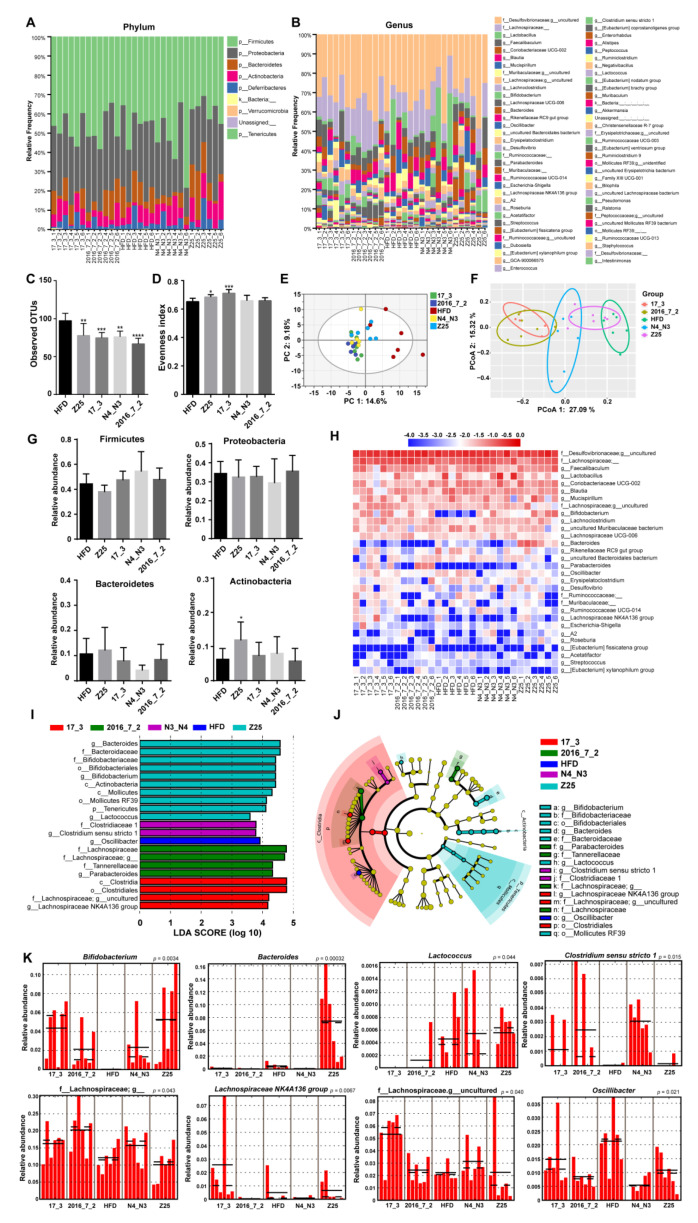
Effects of *B. adolescentis* supplementations on cecal gut microbiota. (**A**,**B**) Microbiota composition by phylum and genus. (**C**,**D**) Alpha diversity indices of gut microbiota, (**C**) observed operational taxonomic units (OTUs), and (**D**) Evenness. (**E**) Principal component analysis (PCA) score scatter plots for gut microbiota (PC 1 = 14.6%, PC 2 = 9.18%). (**F**) Principal coordinate analysis (PCoA) of gut microbiota (PCoA 1 = 27.09%, PCoA 2 = 15.32%). (**G**) The relative abundance of gut microbiota by phylum. (**H**) Heatmap of the proportion of top 30 OTUs determined as dominant bacteria. (**I**) Linear discriminant analysis (LDA) scores of gut microbiota. (**J**) Cladograms representing the LDA effect size (LEfSe) results. (**K**) The abundance histograms of biomarkers detected by LEfSe. Mean values ± SDs are plotted. Asterisks indicate significant differences (Kruskal–Wallis pairwise test for figure (**C**,**D**): * *p* < 0.05, ** *p* < 0.01,*** *p* < 0.001, **** *p* < 0.0001; one-way ANOVA for figure (**G**): * *p* < 0.05; *n* = 6 mice per group).

After 14 weeks of the *B. adolescentis* intervention, the gut microbiota composition of the supplementation groups was significantly altered (Figure 4A,B). Alpha diversity analysis showed that the observed operational taxonomic units (OTUs) in the HFD group were notably higher than those in *B. adolescentis* supplementation groups (HFD vs. Z25: *p* = 0.0025; HFD vs. 17_3: *p* = 0.0007; HFD vs. N4_N3: *p* = 0.0012; HFD vs. 2016-7-2: *p* < 0.0001, Figure 4C). In addition, the evenness indices of the *B. adolescentis* Z25 and 17_3 supplementation groups were higher than that of the HFD group (HFD vs. Z25: *p* = 0.0386; HFD vs. 17_3: *p* = 0.0005, Figure 4D). However, there was no difference in the Faith pd and Shannon indices between the HFD group and the *B. adolescentis* treatment groups (data not shown). Beta diversity analysis-based PCA and principal coordinates analysis (PCoA) showed that the distance of the *B. adolescentis* supplementation groups was far from the HFD group, which indicates that the gut microbiota compositions of the *B. adolescentis* supplementation groups were different from that of the HFD group (Figure 4E,F).

The analysis of the intestinal microbiota composition shows that the microorganisms mainly comprised Firmicutes, Proteobacteria, Bacteroidetes, and Actinobacteria (Figure 4A). *B. adolescentis* Z25 treatment significantly increased the relative abundance of Actinobacteria (*p* = 0.0292) compared with that in the HFD group, but the *B. adolescentis* supplementation groups had no effect on the abundance of Firmicutes, Proteobacteria, or Bacteroidetes, or the Firmicutes:Bacteroidetes ratio (data not shown) (Figure 4G). Based on the relative abundance of the gut microbiota in all groups, the top 30 OTUs were selected as dominant bacteria for further analysis, and the relative abundance of these dominant bacteria accounted for more than 95%.

The heatmap in Figure 4H reveals that there was a deficiency of *Bifidobacterium* in the HFD group. In addition, the groups treated with *B. adolescentis* isolated from the elderly had greater relative abundances of *Parabacteroides* than that in the HFD group (Figure 4H), while the *B. adolescentis* Z25 supplementation group contained the highest abundance of *Bacteroides*. The biomarkers of the gut microbiota were identified by ANCOM and LefSe. ANCOM results showed that *Bacteroides*, *Bifidobacterium,* and *Parabacteroides* were identified as differentiate features. LefSe revealed 21 discriminative features, including eight genera from five groups (Figure 4I,J). The eight genera of biomarkers were *Bifidobacterium* (Z25, *p* = 0.0034), *Bacteroides* (Z25, *p* = 0.00032), *Lactococcus* (Z25, *p* = 0.044), *Clostridium sensu stricto 1* (N4_N3, *p* = 0.015), unclassified Lachnospiraceae bacterium (2016_7_2, *p* = 0.043), *Lachnospiraceae NK4A136 group* (17_3, *p* = 0.0067), uncultured Lachnospiraceae bacterium (17_3, *p* = 0.040), and *Oscillibacter* (HFD, *p* = 0.021) (Figure 4K). 

#### 3.4.2. Gut Microbiota Composition in the Colon

**Figure 5:**
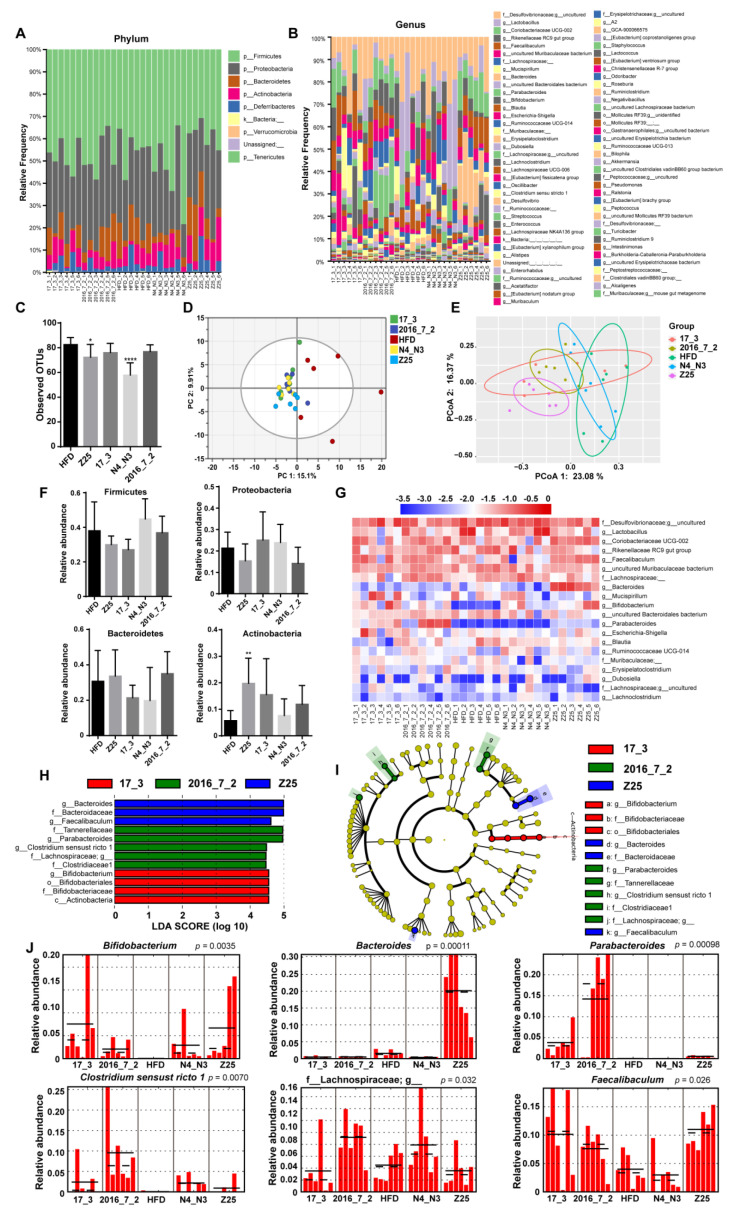
Effects of *B. adolescentis* supplementations on colonic gut microbiota. (**A**,**B**) Microbiota composition by phylum and genus. (**C**) Alpha diversity index observed OTUs, (**D**) PCA score scatter plots for colonic gut microbiota (PC 1 = 15.10%, PC 2 = 9.91%). (**E**) PCoA of colonic gut microbiota (PCoA 1 = 23.08%, PCoA 2 = 16.37%). (**F**) The relative abundance of colonic gut microbiota by phylum. (**G**) Heatmap of the proportion of the top 20 OTUs determined as dominant bacteria. (**H**) LDA scores of colonic gut microbiota. (**I**) Cladograms representing the LEfSe results. (**J**) The abundance histograms of biomarkers detected by LEfSe. Mean values ± SDs are plotted. Asterisks indicate significant differences (Kruskal–Wallis pairwise test for (**C**): * *p* < 0.05, **** *p* < 0.0001; one-way ANOVA for (**F**): ** *p* < 0.01; *n* = 6 mice per group).

The composition of the colonic gut microbiota was also modified by *B. adolescentis* intervention (Figure 5A,B). The alpha diversity analysis results of the colonic gut microbiota were similar to those obtained by an analogous analysis of the cecum microbiota. The observed OTUs of the HFD group were notably higher than those of the *B. adolescentis* Z25 and N4_N3 supplementation groups (HFD vs. Z25: *p* = 0.0393; HFD vs. N4_N3: *p* < 0.0001, Figure 5C). However, there was no difference in the Evenness, Faith pd, and Shannon indices between the five groups (data not shown). Beta diversity analysis-based PCA and PCoA classified the HFD group and *B. adolescentis* supplementation groups into different clusters and showed that the latter groups were far from the former group (Figure 5D,E).

Interestingly, the microbiota composition of the colon was similar to that of the cecum, comprising mainly Firmicutes, Proteobacteria, Bacteroidetes, and Actinobacteria (Figure 5A,F). *B. adolescentis* Z25 supplementation also increased the relative abundance of Actinobacteria in the colon (*p* = 0.0096) compared with that in the HFD group, while no difference was observed in the relative abundances of Firmicutes, Proteobacteria, and Bacteroidetes between the five groups (Figure 5F). The relative abundance of the top 20 OTUs accounted for more than 95%, and these OTUs were selected for heatmap plotting (Figure 5G), which shows that the *B. adolescentis* intervention corrected the colonic *Bifidobacterium* deficiency induced by the HFD, while the relative abundance of *Parabacteroides* was lower in the HFD group and the *B. adolescentis* N4_N3 supplementation group. In addition, the genus *Bacteroides* was notably more abundant in the *B. adolescentis* Z25 supplementation group than in all of the other groups. Twelve discriminative features were identified as biomarkers by LEfSe, including six genera from three groups (Figure 5H,I). The six genera biomarkers were *Bifidobacterium* (17_3, *p* = 0.0035), *Bacteroides* (Z25, *p* = 0.00011), *Faecalibaculum* (Z25, *p* = 0.026), *Parabacteroides* (2016_7_2, *p* = 0.00098), *Clostridium sensu stricto 1* (2016_7_2, *p* = 0.0070), and f_Lachnospiraceae;g_ (2016_7_2, *p* = 0.032) (Figure 5J). ANCOM results showed that *Bacteroides*, *Bifidobacterium, Parabacteroides,* and *Akkermansia* (Figure S4) were identified as differentiate features.

### 3.5. B. adolescentis Supplementation Alters the Production and Composition of SCFAs in the Gut

**Figure 6:**
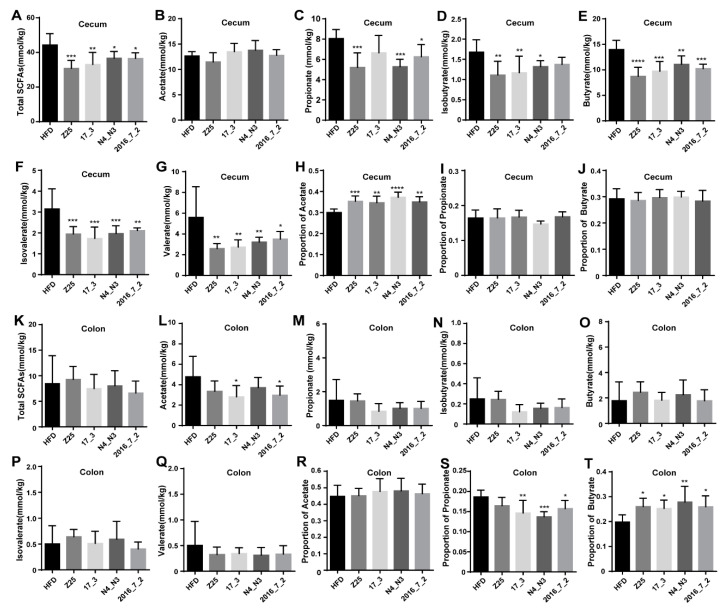
Effects of *B. adolescentis* supplementations on intestinal short-chain fatty acids (SCFAs). (**A**–**G**) Concentrations of cecal SCFAs, (**A**) total SCFAs, (**B**) acetate, (**C**) propionate, (**D**) isobutyrate, (**E**) butyrate, (**F**) isovalerate, and (**G**) valerate. (**H**–**J**) Proportion of cecal SCFAs, (**H**) acetate, (**I**) propionate, and (**J**) butyrate. (**K**–**Q**) Concentrations of colonic SCFAs, (**K**) total SCFAs, (**L**) acetate, (**M**) propionate, (**N**) isobutyrate, (**O**) butyrate, (**P**) isovalerate, and (**Q**) valerate. (**R**–**T**) Proportion of colonic SCFAs, (**R**) acetate, (**S**) propionate, and (**T**) butyrate. Mean values ± SDs are plotted. Asterisks indicate significant differences (one-way ANOVA, * *p* < 0.05, ** *p* < 0.01, *** *p* < 0.001, **** *p* < 0.0001, *n* = 6 mice per group).

*B. adolescentis* supplementation modified the intestinal microbiota and altered the gut SCFA concentrations. According to the food intake of mice, there was no difference in fiber intake between the HFD group and the *B. adolescentis* supplementation groups, except for in the *B. adolescentis* N4_N3 supplementation group. However, although the *B. adolescentis* N4_N3 supplementation group ingested more fiber than the HFD group, the total cecal SCFA concentration of the HFD group was significantly higher than that of all *B. adolescentis* supplementation groups (Figure 6A). Compared with the HFD group, the *B. adolescentis* Z25, N4_N3, and 2016_7_2 supplementation groups had significantly lower concentrations of propionate (Figure 6B), and the *B. adolescentis* Z25, 17_3 and N4_N3 supplementation groups had lower concentrations of isobutyrate in the cecal contents (Figure 6C). All the *B. adolescentis* supplementation groups had lower concentrations of butyrate, isovalerate, and valerate in the cecal tissue compared with the HFD group (Figure 6D–G). Although there was no difference in the concentration of acetate in the cecal contents between any of the groups (Figure 6B), the proportion of acetate in the cecal tissue of the *B. adolescentis* supplementation groups was higher than that in the HFD group (Figure 6H). 

In the colon, there was no significant difference in the concentration of total SCFAs between the groups (Figure 6K). However, the *B. adolescentis* 17_3 and 2016_7_2 supplementation groups exhibited reduced concentrations of acetate in the colonic contents (Figure 6L), although there was no statistically significant difference in the concentrations of propionate, isobutyrate, butyrate, isovalerate, or valerate, or the proportion of acetate, in the colon between the HFD group and the *B. adolescentis* supplementation groups (Figure 6M–R). Interestingly, the proportion of propionate in the colonic tissue of the HFD group was significantly higher than that in the *B. adolescentis* supplementation groups (Figure 6S), while the converse was true for the proportion of colonic butyrate (Figure 6T).

### 3.6. Predicted Functions and Metabolic Pathways in the Gut Microbiota

**Figure 7:**
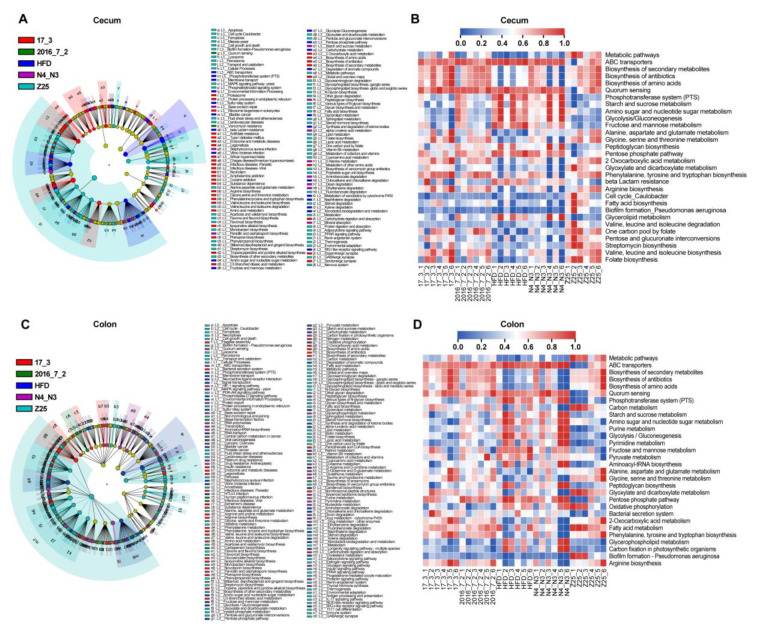
The predicted functions of the intestinal microbiota are altered by the *B. adolescentis* supplementations. (**A**) Cladograms representing the LEfSe results of discriminative KEGG pathways according to the predicted functions of cecal microbiota. (**B**) Heatmap of the relative abundance of the top 30 pathways based on the LEfSe results of cecal microbiota. (**C**) Cladograms representing the LEfSe results of discriminative KEGG pathways according to the predicted functions of colonic microbiota. (**D**) Heatmap of the relative abundance of the top 30 pathways based on the LEfSe results of colonic microbiota.

*B. adolescentis* supplementation significantly altered the cecal and colonic microbiota composition, and the concentration of SCFAs in these tissues. Therefore, the functions of the intestinal microbiota were further studied. The 16S rRNA gene sequencing data of the intestinal microbiota were analyzed using Tax4fun2 to predict functional profiles. The predicted functional profiles show that 298 pathways in the cecal microbiota are related to the gut microbiota. Furthermore, LEfSe analysis of the predicted pathways revealed that 125 pathways were discriminative (Figure 7A). According to the relative abundance of predicted pathways, the 30 most abundant pathways were selected for further analysis (Figure 7B). The relative abundance of pathways predicted to be involved in the biosynthesis of secondary metabolites, antibiotics, and amino acids in the HFD group were much lower than that in the *B. adolescentis* supplementation groups. However, the abundance of pathways with predicted functions in carbohydrate metabolism and membrane transport, such as amino sugar and nucleotide sugar metabolism, ABC transporters, fructose and mannose metabolism, glycolysis/gluconeogenesis, starch and sucrose metabolism, and the phosphotransferase system, was greater in the HFD group and the *B. adolescentis* N4_N3 supplementation group. In addition, the predicted profiles showed that the abundance of the beta-lactam resistance pathway was the highest in the HFD group, which indicates that *B. adolescentis* treatment decreased the risk of HFD-induced antibiotic resistance. 

The predicted functional profiles of the colonic microbiota revealed that they are associated with 306 predicted pathways, and LEfSe analysis showed that there were 175 pathways with discriminative features in the five groups (Figure 7C). A heatmap shows the relative abundance of the top 30 pathways (Figure 7D). Interestingly, the predicted profiles of the top 30 pathways in the colon were similar to those in the cecum. The *B. adolescentis* supplementation groups exhibited a significant increase in pathways related to the biosynthesis of secondary metabolites, antibiotics, and amino acids, while the HFD group and *B. adolescentis* N4_N3 supplementation group exhibited a significant increase in pathways associated with carbohydrate metabolisms, such as pathways involved in the metabolism of amino sugars, nucleotide sugars, fructose, mannose, pyruvate, pentose phosphate, starch, and sucrose. Besides, the abundance of pathways associated with glycolysis/gluconeogenesis, the phosphotransferase system, and purine and pyrimidine metabolism in the HFD group and the *B. adolescentis* N4_N3 supplementation group was higher than that in the other *B. adolescentis* supplementation groups. 

## 4. Discussion

An HFD is known to significantly increase the body weight of mice, which causes obesity, blood glucose impairment, inflammation, and gut microbiota dysbiosis [21,22], while probiotic supplementation can alleviate metabolic disorders and improve energy metabolism [11]. *B. adolescentis* is commonly found in human intestines and plays a role in the regulation of immunity and energy metabolism [14,23]. In this study, HFD-fed mice received *B. adolescentis* interventions, and it was found that *B. adolescentis* strains isolated from the feces of newborn and elderly humans had diverse effects on the improvement of HFD-induced obesity. One *B. adolescentis* improved the blood glucose, and all *B. adolescentis* isolated from the elderly increased energy metabolism in the BAT and corrected the *Bifidobacterium* deletion induced by the HFD, as well as increasing the abundance of beneficial commensal bacteria in the gut. Besides, treatment with *B. adolescentis* strains inhibited inflammation induced by the HFD in mice.

After 14 weeks of treatment with an HFD and *B. adolescentis* N4_N3 (isolated from the feces of the newborn), mice exhibited a significantly increased food intake and RER compared with the HFD-fed mice. Thus, the treated mice gained more energy and metabolized less fat, which led to excessive fat accumulation. In contrast, after 14 weeks of treatment with an HFD and any of the *B. adolescentis* isolated from the feces of elderly humans, mice exhibited significantly decreased body weight or weight gain compared with the HFD-fed mice, but no decrease in food intake. *B. adolescentis* 2016_7_2 treatment also decreased the RER of mice, showing that they consumed more lipids for energy metabolism than the HFD-fed mice.

The activation of PPAR-γ is known to trigger the differentiation of brown adipocytes and adipogenesis, and the activation of PPAR-α induces the terminal differentiation of adipocytes from fibroblasts [24]. In addition, high PPAR-α expression promotes the differentiation of white fat to brown fat [25], and both PPAR-α and PPAR-γ can activate PGC-1α and induce the activation of UCP-1 in the BAT [26]. Similarly, in the current study, *B. adolescentis* isolated from the feces of elderly humans upregulated the mRNA expression of the thermogenesis-related genes *Ucp-1*, *Pgc1-α*, *Ppar-γ*, and *Ppar-α*, and the mRNA expression of lipolytic enzymes, to produce fatty acids for use in non-shivering thermogenesis in the BAT. In the liver, the mRNA expression of *Ppar-γ* and *Fasn* were reduced by *B. adolescentis* Z25. Additionally, treatment with *B. adolescentis* N4_N3 upregulated the expression of *Ppar-γ* in the sWAT and increased the mass of aWAT, while treatment with *B. adolescentis* Z25 and 17_3 showed a tendency to decrease the mRNA expression of *Ppar-γ* and the mass of aWAT, which indicated that different *B. adolescentis* strains might play various roles in lipid accumulation. Overall, *B. adolescentis* treatment strain-dependently activated the mRNA expression of thermogenesis and lipidolysis genes, which might promote lipid metabolism in the BAT, rather than in the liver or sWAT. Thus, we deemed that strain-dependent *B. adolescentis* supplementation could play a role in energy metabolism and the improvement of obesity.

The activation of FXR or TGR5 can induce adipose tissue browning [17,27]. Treatment with *B. adolescentis* isolated from the feces of elderly humans significantly upregulated the expression of *Tgr5* mRNA in the ileum, but not that of *Fxr* mRNA. Activation of TGR5-induced GLP-1 release from L cells promotes adipose browning [28]. The secretion of GLP-1 is dependent on food consumption and it is rapidly degraded, due to its short half-life of approximately 2 min. Although treatment with the *B. adolescentis* isolated from elderly humans upregulated the expression of ileal Tgr 5 and proglucagon gene Gcg mRNA, the fasting concentrations of GLP-1 in serum were low in all groups. In the BAT, *B. adolescentis* interventions did not increase the expression of *Tgr5* mRNA (data not shown); thus, we speculate that the activation of thermogenesis genes was not induced by *Tgr5*.

Leptin is a hormone that regulates appetite and energy homeostasis and increases UCP-1 expression and energy expenditure in the BAT [29]. In this study, we found that *B. adolescentis* isolated from the feces of elderly humans significantly increased serum leptin concentrations, consistent with the expression of *Ucp-1*, *Pgc-1α*, *Ppar-γ*, and *Hsl* mRNA in the BAT. Therefore, we speculate that increased concentrations of leptin might induce BAT browning and lipolysis, and thus suppress weight gain.

HFD-induced obesity is characterized by chronic low-grade inflammation, which is linked to metabolic and non-metabolic diseases [9,30]. Interestingly, we found that *B. adolescentis* supplementation alleviated inflammation of the spleen and brain of the mice. A 14-week HFD-induced splenomegaly in mice and *B. adolescentis* treatment reversed this status via various signaling pathways related to the immune response. 

FOXP3 is a key transcription factor that regulates regulatory T cells (Treg) thereby controlling the immune response. Interleukin 10 (IL-10) is an anti-inflammatory cytokine that inhibits Th17 cell-mediated inflammation. IL-10 activates Treg cells to suppress pathogenic Th17 cell-mediated responses [31], and IL-10 produced by Treg cells controls Th17 cell-mediated inflammation in tumors [32]. *B. adolescentis* strains have various immunoregulatory abilities related to Treg and Th17 cell differentiation and cytokine secretion [23]. In this study, *B. adolescentis* 2016_7_2 treatment significantly upregulated the expression of *Foxp3* and *Il-10* mRNA in mice, and inhibited the expression of *Il-17* mRNA, indicating that *B. adolescentis* 2016_7_2 may activate the regulatory pathway of the IL10-Treg/Th17 axis. *B. adolescentis* Z25 and *B. adolescentis* 17_3 treatment increased the expression of mRNA of the anti-inflammatory cytokine gene *Il-4*, and *B. adolescentis* N4_N3 treatment increased the expression of *Foxp3* mRNA, which would have protected mice from inflammation due to splenomegaly. Furthermore, the analysis of cytokine concentrations in the brains of the mice indicated that all four *B. adolescentis* strains effectively protected the mice from inflammation. All of the *B. adolescentis* strains decreased the secretion of the inflammatory cytokine IL-6 in the brain. In addition, *B. adolescentis* Z25 treatment induced the secretion of IL-10 against a high concentration of IL-17, while treatment with other *B. adolescentis* strains maintained a low concentration of IL-17 in the brain. All *B. adolescentis* strains induced *Foxp3* gene expression and inhibited *Il-6* and *Tlr4* expression in the hypothalamus. TLR4 recognizes lipopolysaccharide (LPS) and activates the NF-κB pathway, in addition to inducing inflammatory cytokine secretion [33]. *B. adolescentis* treatment can reduce intestinal permeability and improve the intestinal barrier to reduce LPS passage across the intestinal barrier and suppress the NF-κB pathway and IL-6 generation [14,34]. In this study, all the *B. adolescentis* treatments alleviated tissue inflammation, which proves that *B. adolescentis* treatment is a feasible approach for regulating immunity, although more work must be done to develop practical applications.

An HFD induces gut microbiota dysbiosis and decreases the abundance of *Bifidobacterium* [10,16]. In this study, it was found that *B. adolescentis* treatment corrected HFD-induced *bifidobacteria* deficiency in the gut microbiota of the cecum and colon. The abundance of *Oscillibacter* was significantly increased in HFD-fed mice without *B. adolescentis* treatment, which was consistent with the findings of previous studies [16,35]. *Oscillibacter* is able to produce SCFAs and medium-chain fatty acids [36,37], which provide additional energy sources for the HFD-fed mice. *Bacteroides* is a dominant genus in the human intestine and plays key roles in maintaining the stability of the gut microbiota, host immunity, and energy metabolism, possessing enormous potential to be a next-generation probiotic [38,39,40]. In this study, treatment with *B. adolescentis* Z25 notably increased the relative abundance of *Bacteroides* in both cecal and colonic microbiota, consistent with the study of Vanessa et al. which showed that co-housing mice receiving the microbiota from an obese twin with mice receiving the microbiota from a lean twin increased the abundance of *Bacteroides* and prevented the former from developing obesity [41]. Therefore, we speculated that the increased *Bacteroides* in the *B. adolescentis* Z25 group might play a role in alleviating HFD-induced obesity. 

*Parabacteroides* is a commensal bacterium in the gut that is negatively related to obesity [42,43,44,45] and plays a role in anti-inflammatory processes [46,47]. Treatment with *Parabacteroides distasonis* has been reported to ameliorate metabolic dysfunction and alleviate obesity in HFD-fed mice [48]. In this study, *B. adolescentis* 17_3 and *B. adolescentis* 2016_7_2 were found to significantly increase the abundance of *Parabacteroides* in the cecum and colon, indicating that these two strains cooperated with *Parabacteroides* to protect the mice from the impairment caused by the HFD. Furthermore, gut microbial genera such as *Bifidobacterium*, *Bacteroides*, *Parabacteroides,* and *Lactobacillus* can deconjugate and alter the composition of bile acids to regulate energy metabolism and immunity [48,49]. In this study, ileal expression of *Tgr5* mRNA was notably increased by supplementation with *B. adolescentis* isolated from the feces of elderly humans, indicating that the gut microbiota modified by these strains might alter the composition of bile acids and activate the bile-acid receptor TGR5. In addition, an HFD has been shown to significantly reduce the abundance of *Faecalibaculum* [12,16], a genus that possesses anti-inflammatory and antitumor properties [50,51]. In this study, treatment with *B. adolescentis* isolated from the feces of elderly humans increased the abundance of *Faecalibaculum* in mice, which might facilitate energy metabolism and anti-inflammatory processes, consistent with a previous study of probiotic supplementation.

Intestinal SCFAs are metabolites of the gut microbiota that are mainly produced in the cecum and colon and are important energy sources for hosts. In this study, we found that *B. adolescentis* treatment reduced the production of intestinal SCFAs in mice, especially in the cecum. This is attributable to the richer nutrient environment in the cecum than that in the colon. Interestingly, the cellulose intake of the *B. adolescentis* N4_N3 supplementation group was higher than that of the HFD group, but the production of SCFAs in the *B. adolescentis* N4_N3 group was lower. This indicates that the cecal microbiota in the HFD group had a higher rate of cellulose utilization than those in the *B. adolescentis* supplementation groups, which generated more energy for mice, leading to fat accumulation.

*B. adolescentis* Z25, 17_3, and 2016_7_2 were isolated from the feces of various elderly humans over the age of 90, while *B. adolescentis* N4_N3 was isolated from the feces of a newborn human who was born by cesarean section and fed breast milk and formula milk. Both kinds of milk are rich in lactose, galactose, and carbohydrates, which favors colonization by *Bifidobacterium*, including *B. adolescentis*. In another study, we found the *B. adolescentis* strain isolated from the feces of another newborn human also increased the food intake and body weight of mice fed the HFD (data not shown). The metabolic rates of newborns are much higher than those of the elderly, and thus, newborns can greatly increase their utilization of food to satisfy their need for growth. We found that compared with *B. adolescentis* strains isolated from the feces of the elderly, *B. adolescentis* N4_N3-treated mice had enhanced energy and their gut microbiota contained a greater abundance of genes involved in carbohydrate metabolism and membrane transport. This implies that intestinal microbiota modified by *B. adolescentis* N4_N3 treatment might offer more energy for mice [12].

The predicted functional profiles showed that *B. adolescentis* isolated from the feces of the elderly modified the gut microbiota such that it might consume more fat, and increased the activity of genes involved in thermogenesis, which was consistent with the analysis of thermogenesis in the BAT. Studies have been proved that probiotics *Lactobacillus paracasei* NCC2461 and *Lactobacillus Reuteri* 263 are able to reduce abdominal fat weight and activate adipose tissue thermogenesis [52,53]. According to the results of energy intake and the gut microbiota, we speculate that the *B. adolescentis* N4_N3 from the newborn may promote appetite and thus offer the newborn more energy from carbohydrate digestion, while *B. adolescentis* and the gut commensal bacteria from the elderly maintain energy homeostasis and keep elderly people lean. Lu et al. found that the gut microbiota in Sardinian centenarians displayed an enrichment in *B. adolescentis* and a deficiency in genes encoding enzymes of carbohydrate degradation than the elders and young people [54]. Thus, the metabolic statuses of newborns and the elderly are different, and different gut microbiota compositions are generated to satisfy their needs. This implies that *B. adolescentis* strains isolated from different people might have inherited the metabolic functions of their original hosts, such that the treatment of mice with *B. adolescentis* isolated from different people may induce different metabolic statuses, even in mice fed the same diet, which is consistent with Jeffrey Gordon’s perspective that microbiota can transmit donor metabotypes to the receptors [41]. In addition, *B. adolescentis* in humans plays an important role in immunoregulation and is beneficial for the host, and thus colonizes the human gut [55,56]. *B. adolescentis* supplementation increased the abundance of beneficial commensal bacteria of mice, which might maintain intestinal health and stable intestinal microbiota composition.

*B. adolescentis* is a dominant genus in the healthy human intestine and is known as a probiotic. Our previous studies found that *B. adolescentis* supplementations can decrease the intestinal permeability and alleviate liver injury in nonalcoholic fatty liver disease (NAFLD) and in metabolic diseases of rodents [14,15]. *B. adolescentis* can also specifically induce intestinal Th17 cell accumulation to regulate immunity without attendant inflammation [57]. Additionally, *Bifidobacterium adolescentis* supplementation is able to attenuate fracture-induced systemic sequelae, augment the tightening of the intestinal barrier, inhibit the systemic inflammatory response to fracture, accelerate fracture callus cartilage remodeling, and enhance the protection of the intact skeleton [58]. Besides, *Bifidobacterium adolescentis* supplementation can promote post-antibiotic ecological recovery in the gut, raising the microbial abundance at early time points inducing the faster maturation of microbial diversity to protect the hosts from the long-term consequences of frequent antibiotic usage [59]. Thus, the supplementation of *Bifidobacterium adolescentis* will have a profound impact on host physiology and offer protection for the health of hosts. 

## 5. Conclusions

This study demonstrated that *B. adolescentis* supplementation can alleviate HFD-induced obesity and modify the intestinal microbiota composition, which is accompanied by a reduction in energy delivery to the host due to decreased gut SCFA concentrations. *B. adolescentis* strains isolated from the feces of newborn and elderly humans display different effects on energy metabolism and immunity, which suggests that *B. adolescentis* from different people and regions may play different roles in human health. An in-depth understanding of the characteristics of the *B. adolescentis* strains themselves, and their synergies with commensal bacteria, will help us to select strains for further therapeutic use.

## Figures and Tables

**Table 1:** *B. adolescentis* strain information.

*B. adolescentis* Strain	Sample	Age	Location
HuNan17_3	Human feces	90	Boai, Henan
HuNan2016_7_2 (CCFM1062)	Human feces	93	Boai, Henan
Z25 (CCFM8630)	Human feces	95	Zhongxiang, Hubei
FSDJN4_N3 (CCFM1061)	Human feces	Newborn	Jinan, Shandong

## Data Availability

The data presented in this study are available on request from the corresponding author. The data are not publicly available as the related strains are in the process of patent application.

## Supplementary Materials

The following are available online at https://www.mdpi.com/2072-6643/13/3/1017/s1, Figure S1: Metabolic rates of mice fed a high-fat diet with or without *B. adolescentis* supplementation, Figure S2: Effects of *B. adolescentis* supplementations on energy metabolism, Figure S3: Effects of *B. adolescentis* supplementations on immunity, Figure S4 Relative abundance of Akkermansia in the colon. Table S1. The diet composition of the high-fat diet (TP 23300) 1. Table S2. Primers for real-time PCR analysis of gene expression.
