# Supplementary material for: Bifidobacterium adolescentis Isolated from Different Hosts Modifies the Intestinal Microbiota and Displays Differential Metabolic and Immunomodulatory Properties in Mice Fed a High-Fat Diet
Source: Nutrients. 2021 Mar 21;13(3):1017. doi: 10.3390/nu13031017 (PMC8004121; doi:10.3390/nu13031017)
Supplement: Supplementary file 1 [file nutrients-13-01017-s001.pdf]

Supplementary Materials: The following are available online at www.mdpi.com/xxx/s1, Figure S1: Metabolic rates of mice fed a high-fat diet with or without *B. adolescentis* supplementation, Figure S2: Effects of *B. adolescentis* supplementations on energy metabolism, Figure 3: Effects of *B. adolescentis* supplementations on immunity, Table S1: Primers for real-time PCR analysis of gene expression.

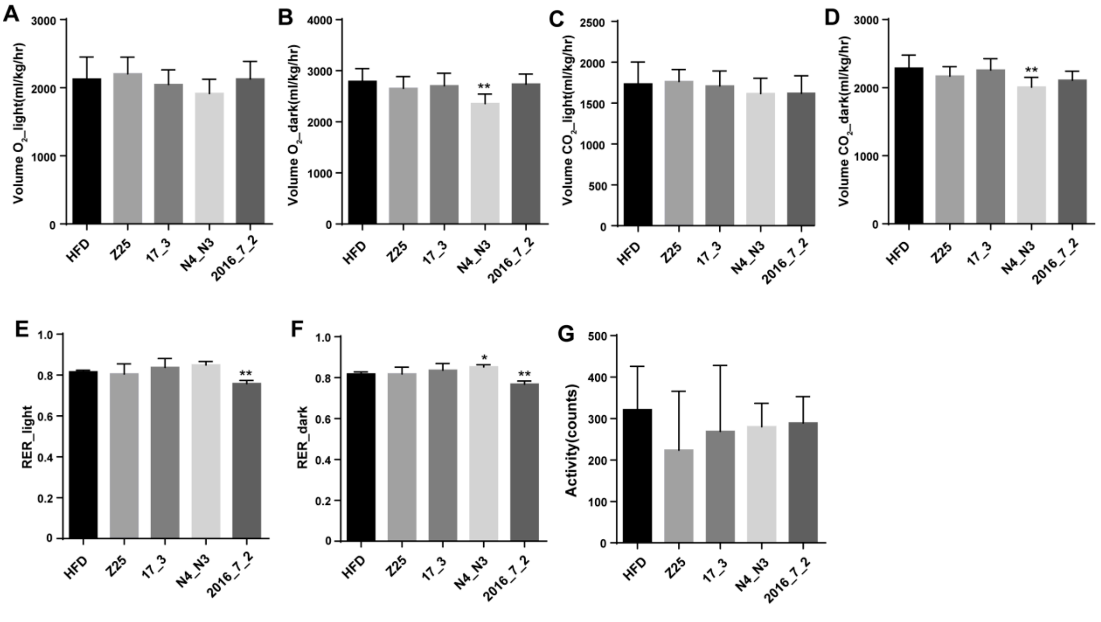

Figure S1: Metabolic rates of mice fed a high-fat diet with or without *B. adolescentis* supplementation. (**A, B**) Consumption of O_2 in the light and dark. (**C,D**) Production of CO_2 in the light and dark. (**E, F**) Respiratory exchange ratio (RER) in the light and dark. (**G**) Activities of mice in 5 groups. Data are shown as means ± standard deviations (SD). Asterisks indicate significant differences (one-way ANOVA, * $p < 0.05$, ** $p < 0.01$,). n = 6 mice per group.

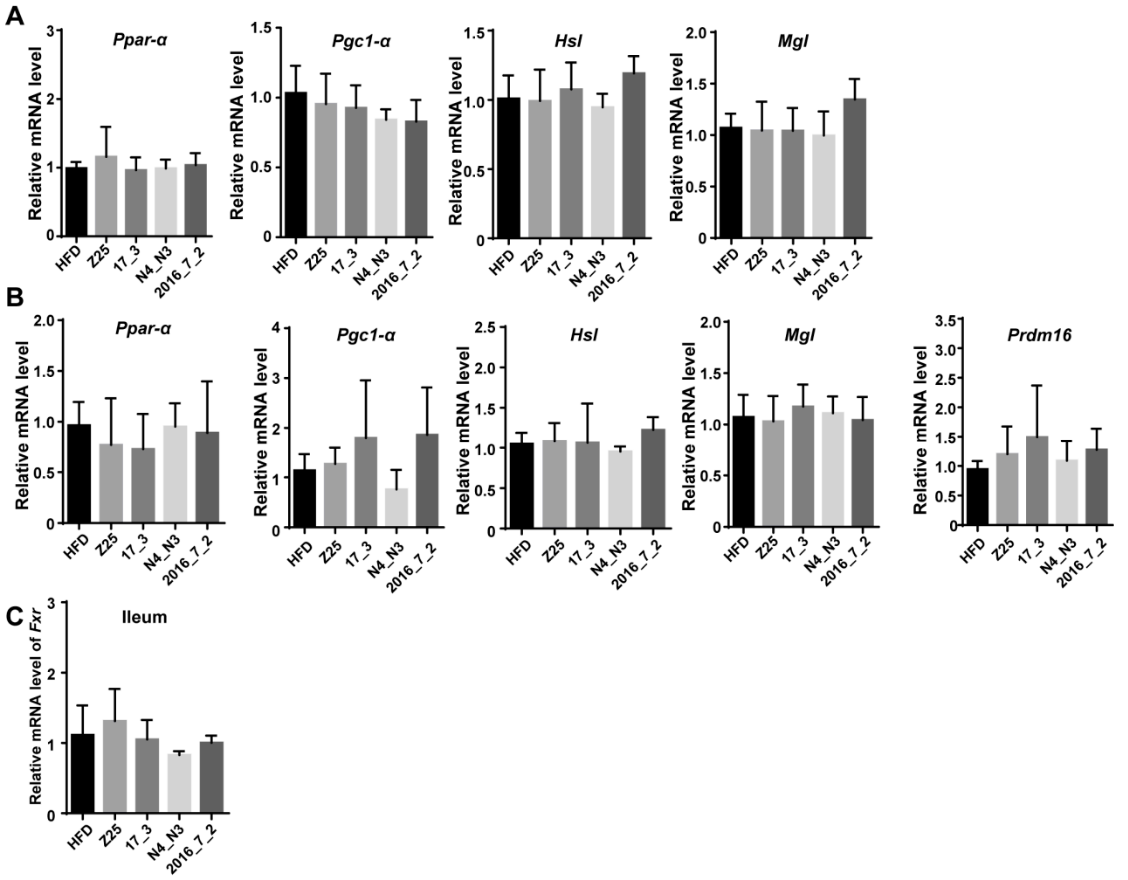

Figure S2: Effects of *B. adolescentis* supplementations on energy metabolism. (**A**) Relative mRNA expression of thermogenesis related genes and lipolytic enzyme genes in the liver. (**B**) Relative mRNA expression of thermogenesis related genes and lipolytic enzyme genes in the subcutaneous white adipose tissue (sWAT). (**C**) Relative mRNA expression of bile acid receptor farnesoid X receptor (*Fxr*) in the ileum. Data are shown as means ± standard deviations (SD). n = 5 mice per group.

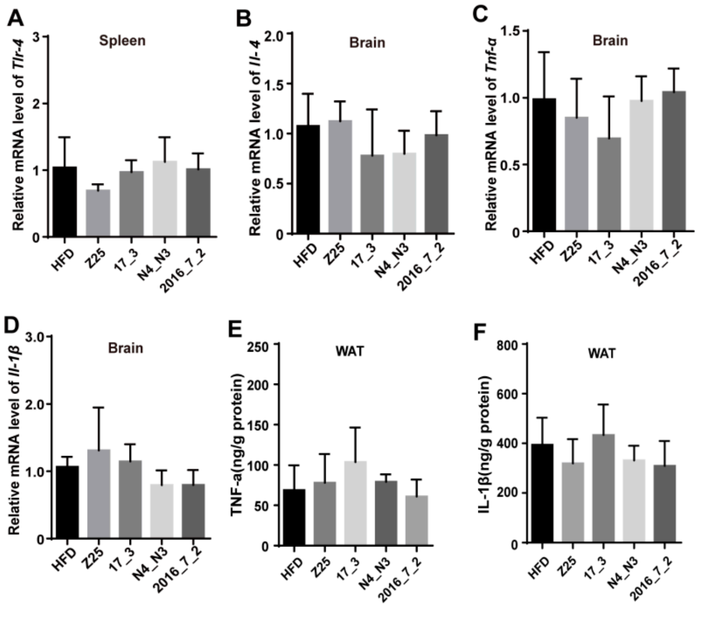

Figure S3: Effects of *B. adolescentis* supplementations on immunity. (**A**) Relative mRNA expression of immune-related toll-like receptor 4 (*Tlr4*) in the spleen. (**B-D**) Relative mRNA expression of immune-related receptor and cytokines in the hypothalamus, (B) interleukin 4 (*Il-4*), (**C**) tumor necrosis factor α (*Tnf-α*), and (**D**) *Il-1β*. (**E, F**) Concentrations of cytokines in the abdominal white adipose tissue (aWAT), (**E**) TNF-α, and (**F**) IL-1β. Data are shown as means ± standard deviations (SD). n = 6 mice or n = 5 (for A-D) per group.

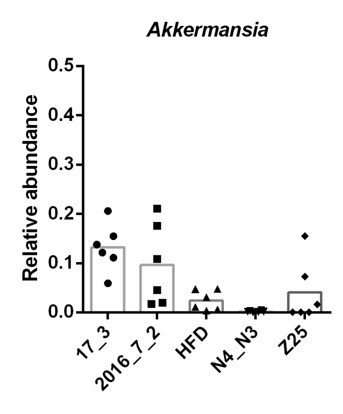

Figure S4 Relative abundance of *Akkermansia* in the colon

Table S1. The diet composition of the high-fat diet (TP 23300) [1]

Ingredient, g/kg	TP 23300
Casein	259
Maltodextrin	166
Sucrose	91
Corn Starch	0
Soybean Oil	33
Lard	313
Cellulose	67
Mineral Mix, M1020	52
Vitamin Mix, V1010	13
L-Cystine	3
Choline Bitartrate	3
TBHQ	0.067
Total	1000.067

1. The purified diet TP 23300 supplies energy as 20% carbohydrate (7% sucrose calories), 20% protein, 60% fat with total 5.1 kcal/g.

Table S2. Primers for real-time PCR analysis of gene expression[1].

Gene	PrimerBank ID	Forward Primer	Reverse Primer
Ucp1	6678497a1	AGGCTTCCAGTACCATTAGGT	CTGAGTGAGGCAAAGCTGATTT
Pgc1-α	6679433a1	TATGGAGTGACATAGAGTGTGCT	CCACTTCAATCCACCCAGAAAG
Ppar-α	31543500a1	AGAGCCCCATCTGTCCTCTC	ACTGGTAGTCTGCAAAACCAAA
Ppar-γ	6755138a1	TCGCTGATGCACTGCCTATG	GAGAGGTCCACAGAGCTGATT
Fasn	30911099a1	GGAGGTGGTGATAGCCGGTAT	TGGGTAATCCATAGAGCCCAG
Hsl	26325924a1	CCAGCCTGAGGGCTTACTG	CTCCATTGACTGTGACATCTCG
Mgl	6754690a1	CGGACTTCCAAGTTTTTGTCAGA	GCAGCCACTAGGATGGAGATG
Fxr	254911038c1	GGCAGAATCTGGATTTGGAATCG	GCCCAGGTTGGAATAGTAAGACG
Tgr5	27923942c2	CTGTGTGAGATCCGCCGAC	CGACGCTCATAGGCCAAGA
Prdm16	124107622c3	CCCCACATTCCGCTGTGAT	CTCGCAATCCTTGCACTCA
Il-17f	22003916a1	TGCTACTGTTGATGTTGGGAC	AATGCCCTGGTTTTGGTTGAA
Tnf-α	133892368c2	CTGAACTTCGGGGTGATCGG	GGCTTGTCACTCGAATTTTGAGA
Tlr4	10946594a1	ATGGCATGGCTTACACCACC	GAGGCCAATTTTGTCTCCACA
Il-6	13624311a1	TAGTCCTTCCTACCCCAATTTCC	TTGGTCCTTAGCCACTCCTTC
Il-4	10946584a1	GGTCTCAACCCCCAGCTAGT	GCCGATGATCTCTCTCAAGTGAT
Il-10	6754318a1	GCTCTTACTGACTGGCATGAG	CGCAGCTCTAGGAGCATGTG
Il-1β	118130747c1	GAAATGCCACCTTTTGACAGTG	TGGATGCTCTCATCAGGACAG
Foxp3	16905075a1	CCCATCCCCAGGAGTCTTG	ACCATGACTAGGGGCACTGTA
Gapdh	6679937a1	AGGTCGGTGTGAACGGATTTG	TGTAGACCATGTAGTTGAGGTCA

[1]. The validated primers were obtained from Primerbank.
